# Association of Lower Third Molar Position and Angulation With Mandibular Anterior Crowding in the Chhattisgarh Population: A Retrospective Radiographic Study

**DOI:** 10.7759/cureus.112791

**Published:** 2026-07-16

**Authors:** Ardhra C A, Virendra Vadher, Shweta Singh, Arvind Nair, Chhaya Barapatre, Gangesh Bahadur Singh

**Affiliations:** 1 Department of Orthodontics and Dentofacial Orthopaedics, Government Dental College and Hospital, Raipur, IND

**Keywords:** ganss ratio, mandibular anterior crowding, mandibular third molar, orthodontics, panoramic radiography, retromolar space, retrospective study, tooth angulation

## Abstract

Introduction

The association between mandibular third molars and mandibular anterior crowding remains controversial. Although several studies have investigated the relationship between third molar characteristics and lower anterior crowding, the relative influence of third molar position and angulation remains inconclusive. This study aimed to evaluate the association between lower third molar position, angulation, and mandibular anterior crowding in a Chhattisgarh population using panoramic radiographs and mandibular study models.

Materials and methods

This retrospective observational study included 234 pretreatment orthodontic records comprising panoramic radiographs and mandibular study models. Subjects aged 16-23 years with Angle's Class I molar relationship and bilaterally present mandibular third molars were allocated into a crowded group (n = 117) and a non-crowded group (n = 117). Mandibular anterior crowding was assessed using pretreatment mandibular study models. Third molar position was evaluated using the retromolar space-to-third molar crown width ratio (Ganss ratio), while third molar angulation was assessed using the inclination between the mandibular second and third molars (Angle A) and the angulation of the mandibular third molar relative to the mandibular base (Angle B). Intergroup comparisons were performed using the independent Student's t-test, and a p-value of <0.05 was considered statistically significant.

Results

The crowded group demonstrated significantly lower Ganss ratios than the non-crowded group on both the right (p = 0.0003) and left (p = 0.0031) sides. Angles B were significantly lower in both tails (right: p = 0.030; left: p = 0.038), but Angle A was only significant on the left (p = 0.0006). The right-sided Angle A had no statistically significant difference (p = 0.349). In general, the third molar position showed a more reliable relationship with mandibular anterior crowding compared to angular measurements.

Conclusion

Lower third molar position and angulation were both associated with mandibular anterior crowding. One of the radiographic measures, the Ganss ratio, showed the lowest variance of association, implying that the available retromolar space measure may offer more diagnostic utility than the angular measurements. These radiograph parameters can be used as helpful adjuncts, but not to be the basis of the prophylaxis of the mandibular third molar extraction in orthodontic diagnoses, as well as treatment planning.

## Introduction

One of the most common malocclusions in orthodontic practice is mandibular anterior crowding, as it has one of the highest levels of referrals to orthodontic care. Besides undermining or compromising the esthetics of the teeth, crowding can lead to a decline in the level of plaque control, periodontal health, and long-term stability of the orthodontic treatment results [1]. Mandibular anterior crowding is generally believed to be a multifactorial phenomenon that is affected by the mismatch of tooth size and arch length, craniofacial development, physiological mesial movement of teeth, occlusal forces, soft tissue pressures, and maturation changes during age [1,2].

The role of mandibular third molars in lower anterior crowding has been one of the most controversial topics of orthodontics. For many years, erupting or impacted mandibular third molars were believed to exert mesially directed forces that could contribute to irregular alignment of the mandibular incisors, providing a rationale for prophylactic removal of asymptomatic third molars [3]. However, subsequent investigations have produced conflicting findings, and the precise role of mandibular third molars in the development of anterior crowding continues to be controversial [4,5].

Several clinical and radiographic studies have investigated the relationship between mandibular third molar characteristics and lower anterior crowding. Reduced retromolar space, inadequate eruption space, and unfavorable third molar angulation have been reported to be associated with an increased prevalence of mandibular anterior crowding [6-10]. Conversely, other investigators have suggested that mandibular third molars are only one of several contributing factors and are unlikely to represent the primary etiological factor responsible for lower incisor crowding [11-13]. Furthermore, systematic reviews and meta-analyses have concluded that the current body of evidence is inconsistent and does not support routine prophylactic extraction of asymptomatic mandibular third molars solely for the prevention of mandibular anterior crowding [4-5,14]. Recent investigations utilizing cone-beam computed tomography (CBCT) have continued to evaluate this association, emphasizing that further high-quality studies are required before definitive conclusions can be drawn [15-17].

Panoramic radiography remains a practical and widely accepted diagnostic tool for evaluating mandibular third molars because it enables simultaneous assessment of tooth position, angulation, and the available retromolar space. Radiographic measurements such as retromolar space to third molar crown width ratio (Ganss ratio), inclination between third molar and second molar in the mandible, and angulation of third molar to that of the mandibular base have been objective radiographic parameters to measure eruption potential and position of the third molar [6-8,18]. These measurements are a set of useful data that could help understand the relationship between mandibular third molars and lower anterior crowding better.

Although the role of mandibular third molars in mandibular anterior crowding has been studied extensively in the last several decades, the effect these molars have has been inconclusive to date. In addition, evidence from the Indian population is limited, and studies evaluating this relationship in the Chhattisgarh population are lacking [4-5,14]. Therefore, the present retrospective observational study was undertaken to evaluate the association between lower third molar position, angulation, and mandibular anterior crowding in the Chhattisgarh population using pretreatment mandibular study models and panoramic radiographs.

## Materials and methods

Study design and sample selection

This retrospective observational study was conducted over a period of six months using archived pretreatment orthodontic records obtained from the Department of Orthodontics and Dentofacial Orthopaedics, Government Dental College, Raipur, following approval from the Institutional Ethics Committee (Approval No. ECR/04/GDC/CG/2025). The minimum sample size was calculated with G*Power software version 3.1 (Heinrich Heine University, Düsseldorf, Germany) according to the results of Hasegawa et al. [7] with the assumptions of the effect size of 0.3689, level of significance (0.05), and statistical power of 80, which indicates the minimum sample size of 234 subjects.

Retrospective screening of archived pre-treatment records, including panoramic radiographs (orthopantomograms) and matching mandibular study models, was performed. Patient records of patients aged 16 to 23 years of the Class I molar relation of Angle, full permanent dentition, and the mandible having bilaterally occurring third molars were included. Patients who have undergone prior orthodontic treatment, artificial dental crowns, and developmental anomalies that impact the morphology of the crowns were not included in the records, as well as incomplete records of pre-treatment and poor quality of panoramic radiographs.

All archived pretreatment records available during the study period were screened consecutively according to the eligibility criteria. Eligible records fulfilling the inclusion and exclusion criteria were included until the required sample size was achieved. The final study sample consisted of 234 participants, including 117 subjects with mandibular anterior crowding (crowded group) and 117 subjects without mandibular anterior crowding (non-crowded group).

Mandibular anterior crowding was assessed on pretreatment mandibular study models using Little’s Irregularity Index [19]. The linear displacement of the anatomic contact points of the six mandibular anterior teeth was measured in millimeters, and the sum of these five measurements represented the irregularity score. Subjects with increased irregularity scores were included in the crowded group, whereas subjects with normal alignment or minimal irregularity were included in the non-crowded group. All measurements were performed by a single examiner.

Radiographic evaluation

Pretreatment panoramic radiographs were obtained using the same panoramic radiographic machine with standardized patient positioning according to the manufacturer’s instructions. The radiographs were hand-traced on 0.003-inch matte acetate tracing sheets using a 0.3-mm lead pencil over an illuminated viewing box. All radiographic measurements were performed by a single trained examiner under standardized viewing conditions. Anatomical landmarks were carefully identified, and all measurements were reviewed by the same examiner before final data entry to minimize transcription errors.

**Figure 1:**
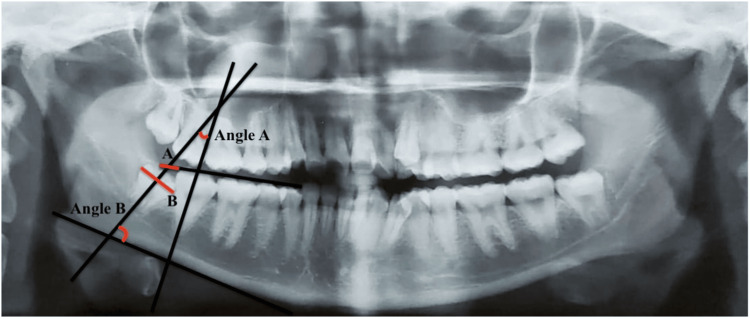
Representative panoramic radiograph illustrating the radiographic measurements used in the study on the right side. (A) Retromolar space. (B) Mesiodistal width of the mandibular third molar crown; Ganss ratio = A/B. Angle A: angle between the long axes of the mandibular second and third molars. Angle B: angle between the long axis of the mandibular third molar and the mandibular base.

The retromolar space-to-third molar crown width ratio (Ganss ratio) was calculated by dividing the retromolar space (A), measured as the distance from the distal surface of the mandibular second molar to the anterior border of the mandibular ramus along the occlusal plane, by the mesiodistal width of the mandibular third molar crown (B) [18]. The angle formed between the long axes of the mandibular second and third molars (Angle A) was measured to determine the second molar-third molar inclination. The angle between the long axis of the mandibular third molar and the mandibular base (Angle B) was also measured to assess third molar angulation relative to the mandibular base. All measurements were obtained separately on the right and left sides (Figure 1).

Statistical analysis

The collected data were entered into Microsoft Excel (Microsoft® Corp., Redmond, WA) and analyzed using the Statistical Package for the Social Sciences (SPSS) software (IBM Corp., Armonk, NY). Continuous variables were expressed as mean ± standard deviation. Intergroup comparisons were performed using the Independent Student's t-test. Welch's correction was applied when the assumption of equal variances was not satisfied. A p-value of <0.05 was considered statistically significant.

## Results

A total of 234 subjects were included in the study, comprising 117 subjects without mandibular anterior crowding (non-crowded group) and 117 subjects with mandibular anterior crowding (crowded group). The mean age of the non-crowded group was 18.09 ± 2.47 years, whereas the crowded group had a mean age of 17.83 ± 2.44 years. The difference in mean age between the groups was not statistically significant (p = 0.425). The non-crowded group included 75 females and 42 males, while the crowded group comprised 85 females and 32 males (Table 1).

**Table 1:** Demographic characteristics of the study population.

Variable	Non-crowded (n = 117)	Crowded (n = 117)
Age (years)	18.09 ± 2.47	17.83 ± 2.44
Male	42	32
Female	75	85

The mean Ganss ratio was significantly greater in the non-crowded group than in the crowded group on both the right (0.80 ± 0.28 vs. 0.67 ± 0.28; p = 0.0003) and left sides (0.81 ± 0.27 vs. 0.68 ± 0.38; p = 0.0031) (Table 2).

**Table 2:** Comparison of Ganss ratio between the non-crowded and crowded groups. An independent Student's t-test was used for intergroup comparison. A p-value of <0.05 was considered statistically significant.

Variable	Non-crowded (mean ± SD)	Crowded (mean ± SD)	t-value	p-value
					Ganss ratio (right)	0.80 ± 0.28	0.67 ± 0.28	3.669	0.0003*
Ganss ratio (left)	0.81 ± 0.27	0.68 ± 0.38	2.992	0.0031*

No statistically significant difference was observed in the right-sided second molar-third molar inclination (Angle A) between the two groups (p = 0.349). However, the left-sided Angle A was significantly greater in the crowded group than in the non-crowded group (34.90° ± 26.43° vs. 24.26° ± 20.16°; p = 0.0006). The third molar angulation relative to the mandibular base (Angle B) was significantly lower in the crowded group than in the non-crowded group on both the right (57.24° ± 22.64° vs. 63.86° ± 24.02°; p = 0.030) and left sides (58.82° ± 19.51° vs. 64.69° ± 23.38°; p = 0.038) (Table 3).

**Table 3:** Comparison of second molar-third molar inclination (Angle A) and third molar angulation to the mandibular base (Angle B) between the non-crowded and crowded groups. An independent Student's t-test was used for intergroup comparison. A p-value of <0.05 was considered statistically significant.

Variable	Side	Non-crowded (mean ± SD)	Crowded (mean ± SD)	t-value	p-value
						Angle A	Right	27.25 ± 22.44	30.16 ± 25.07	-0.937	0.349
Left	24.26 ± 20.16	34.90 ± 26.43	-3.461	0.0006*		
Angle B	Right	63.86 ± 24.02	57.24 ± 22.64	2.172	0.030*
	Left	64.69 ± 23.38	58.82 ± 19.51	2.08	0.038*

## Discussion

The present retrospective observational study evaluated the association between lower third molar position and angulation with mandibular anterior crowding in a Chhattisgarh population. The findings demonstrated significant associations between mandibular anterior crowding and both positional and angular characteristics of the mandibular third molar. However, third molar position, assessed using the Ganss ratio, exhibited a more consistent association with crowding than angular measurements, suggesting that available retromolar space may have greater diagnostic relevance than tooth orientation alone.

The Ganss ratio showed a consistent association with mandibular anterior crowding among the radiographic parameters evaluated in the present study. Subjects with mandibular anterior crowding demonstrated reduced Ganss ratios, indicating decreased available retromolar space in relation to the mesiodistal width of the mandibular third molar crown. The Ganss ratio, which is originally defined by Ganss et al., is a ratio of available eruption space and the size of the third molar crown and has been suggested to indicate eruption potential [18]. The present findings are consistent with Selmani et al., who reported lower Ganss ratios in subjects with mandibular anterior crowding [6]. The same was also observed by Hasegawa et al. and Cherian and Ravi, who showed that decreased retromolar space correlated with poor third molar position and decreased anterior crowding [7-8]. Niedzielska also emphasized the importance of eruption space in determining third molar position and its possible association with lower arch crowding [9]. Moreover, Lakhani et al. indicated that anterior crowding can predict the presence of mandibular third molar impaction, which argues for the correlation between limited eruption space and third molar location [20]. However, mandibular anterior crowding has been considered a multifactorial condition, and the current results suggest that there is an association, but not a direct causal relationship [2-5].

Third molar angulation was evaluated using the inclination between the mandibular second and third molars (Angle A) and the angulation of the mandibular third molar relative to the mandibular base (Angle B). Although Angle B demonstrated significant bilateral differences between the study groups, Angle A was significantly different only on the left side, suggesting that angular measurements were less consistent than positional assessment. Similar findings have been reported by Selmani et al., who observed greater second molar-third molar inclination in subjects with lower anterior crowding [6]. Hasegawa et al. also demonstrated that inadequate retromolar space influences third molar inclination and eruption direction, indicating that tooth angulation is closely related to the available eruption space [7]. Bashir et al. reported a significant association between impacted mandibular third molar angulation and the severity of lower anterior crowding [10]. More recently, CBCT-based studies by Akkitap et al. and Ketenci Çay et al. have further supported the evaluation of third molar angulation when investigating its relationship with mandibular anterior crowding [15,17]. The unilateral significance observed for Angle A in the present study may reflect biological variation in eruption patterns or the limitations of panoramic radiography in representing three-dimensional structures.

When both positional and angular parameters were considered collectively, third molar position demonstrated a more consistent association with mandibular anterior crowding than third molar angulation. This may be explained by the fact that the Ganss ratio incorporates both the available retromolar space and the mesiodistal width of the third molar crown, thereby providing a more comprehensive assessment of eruption space than angular measurements alone [18]. In contrast, angular measurements primarily describe tooth orientation and may be influenced by individual variation in eruption patterns. These findings suggest that evaluation of the retromolar space should complement assessment of third molar angulation during orthodontic diagnosis rather than either parameter being interpreted in isolation.

The present findings are in agreement with systematic reviews by Zawawi and Melis, Genest-Beucher et al., and the recent meta-analysis by Palikaraki et al., which concluded that although mandibular third molars may be associated with lower anterior crowding, the available evidence remains insufficient to establish a direct causal relationship or to justify routine prophylactic extraction of asymptomatic mandibular third molars solely for the prevention of crowding [4-5,14]. Variations in study design, methods of crowding assessment, imaging techniques, and population characteristics may explain the inconsistent findings reported in the literature.

The findings of the present study highlight the importance of evaluating both third molar position and angulation during orthodontic diagnosis and treatment planning. Nonetheless, these radiographic measures are meant to be an addition to the overall clinical examination and to a considerable extent, not relied upon as an isolated measure of prophylaxis mandibular third molar extraction. The strengths of the current study are that the sample size is quite large, the standardized radiographic measurements were used, and both positional and angular parameters were assessed by the same examiner. Nevertheless, the retrospective study design, use of panoramic radiography, absence of intra-examiner reliability assessment for repeated measurements, and inclusion of subjects from a single institution may limit the generalizability of the findings. Further prospective multicenter studies with larger sample sizes and standardized methodologies are recommended to validate these observations.

## Conclusions

Within the limitations of this retrospective observational study, lower third molar position and angulation were both found to be associated with mandibular anterior crowding. Out of the radiographic parameters considered, the Ganss ratio showed the most consistent relationship, indicating that the size of available retromolar space as a proportion of the third molar crown width might be a more accurate predictor of mandibular anterior crowding than angular measurements by themselves. Despite some significant results with angulation of the third molar, the results were not as consistent. The findings validate the application of radiographic evaluation of angulation and position of the third molar as a supplement to the orthodontic diagnosis and treatment planning. Yet decisions related to prophylactic mandibular third molar extraction cannot be made on the basis of these radiographic findings alone, since mandibular anterior crowding is a multifactorial condition influenced by various dental and skeletal factors. Further prospective studies using three-dimensional imaging are required to validate these findings.

## References

[1] Buschang PH, Shulman JD (2003). Incisor crowding in untreated persons 15-50 years of age: United States, 1988-1994. Angle Orthod.

[2] Richardson ME (1994). The etiology of late lower arch crowding alternative to mesially directed forces: a review. Am J Orthod Dentofacial Orthop.

[3] Richardson ME, Orth D (1989). The role of the third molar in the cause of late lower arch crowding: a review. Am J Orthod Dentofacial Orthop.

[4] Zawawi KH, Melis M (2014). The role of mandibular third molars on lower anterior teeth crowding and relapse after orthodontic treatment: a systematic review. ScientificWorldJournal.

[5] Genest-Beucher S, Graillon N, Bruneau S, Benzaquen M, Guyot L (2018). Does mandibular third molar have an impact on dental mandibular anterior crowding? A literature review. J Stomatol Oral Maxillofac Surg.

[6] Selmani ME, Gjorgova J, Selmani ME, Shkreta M, Duci SB (2016). Effects of lower third molar angulation and position on lower arch crowding. Int J Orthod Milwaukee.

[7] Hasegawa Y, Terada K, Kageyama I, Tsuchimochi T, Ishikawa F, Nakahara S (2013). Influence of third molar space on angulation and dental arch crowding. Odontology.

[8] Cherian M, Ravi M S (2016). Lower third molar space and angulation in individuals with lower anterior crowding. J Health Allied Sci.

[9] Niedzielska I (2005). Third molar influence on dental arch crowding. Eur J Orthod.

[10] Bashir S, Nausheen A, Nasir U, Maryam B (2021). Severity of lower anterior arch crowding and angulations of impacted third molar. J Khyber Coll Dent.

[11] Stanaitytė R, Trakinienė G, Gervickas A (2014). Lower dental arch changes after bilateral third molar removal. Stomatologija.

[12] Cotrin P, Freitas KM, Freitas MR, Valarelli FP, Cançado RH, Janson G (2020). Evaluation of the influence of mandibular third molars on mandibular anterior crowding relapse. Acta Odontol Scand.

[13] Sidlauskas A, Trakiniene G (2006). Effect of the lower third molars on the lower dental arch crowding. Stomatologija.

[14] Palikaraki G, Mitsea A, Sifakakis I (2024). Effect of mandibular third molars on crowding of mandibular teeth in patients with or without previous orthodontic treatment: a systematic review and meta-analysis. Angle Orthod.

[15] Akkitap MP (2026). Cone-beam CT evaluation of impacted mandibular third molars and their possible association with mandibular incisor crowding. Med Oral Patol Oral Cir Bucal.

[16] Chansoria A, Gupta Y, Chaudhary V, Mishra K (2025). Role of third molars in anterior crowding: delusion or vindication?. J Contemp Orthod.

[17] Ketenci Çay F, Gül Yılmaz B, Çiğdem Keleş E, İlgüy D (2025). Effect of mandibular third molars on anterior crowding assessed by CBCT. Odovtos Int J Dent Sci.

[18] Ganss C, Hochban W, Kielbassa AM, Umstadt HE (1993). Prognosis of third molar eruption. Oral Surg Oral Med Oral Pathol.

[19] Little RM (1975). The irregularity index: a quantitative score of mandibular anterior alignment. Am J Orthod.

[20] Lakhani MJ, Kadri W, Mehdi H, Sukhia H, Bano A, Yaqoob S (2011). Anterior arch crowding--a possible predictor for mandibular third molar impaction. J Ayub Med Coll Abbottabad.

